# From intention to perception: emotional processes as a link between intended and perceived leadership styles

**DOI:** 10.3389/fpsyg.2025.1526797

**Published:** 2025-05-09

**Authors:** Rosa Mindeguia, Aitor Aritzeta, Alaine Garmendia, Amaiur Olarza

**Affiliations:** ^1^Department of Applied Economics, Faculty of Economics and Business, University of the Basque Country (UPV/EHU), San Sebastian, Spain; ^2^Department of Basic Psychological Processes and Development, Faculty of Psychology, University of the Basque Country (UPV/EHU), San Sebastian, Spain; ^3^Mondragon Unibertsitatea, Department of Mechanic and Industrial Production, Mondragón, Basque Country, Spain

**Keywords:** team emotional intelligence, emotions, intended leadership, perceived leadership, transformational leadership

## Abstract

**Introduction:**

Research has shown that managers and employees often differ in their perceptions of leadership, and that agreement between them is essential for effective leadership. Leadership involves both the actions of leaders and the perceptions of followers who interpret those actions within organizational contexts. Thus, the extent to which intended leadership styles influence followers—and, consequently, organizations—may depend largely on followers’ perceptions. It is therefore important to analyze the relationship between leaders’ intended leadership styles, followers’ perceptions of leadership, and the mediation processes between them. This study explored the mediating effects of management team emotional intelligence (TEI), the discrete emotions of followers and work units, and their roles in linking intended and perceived leadership styles.

**Methods:**

Data were collected from two sources: 1,566 managers organized into 188 teams, and 4,564 workers. Multilevel path analysis was used to examine the relationships among variables.

**Results:**

The findings showed that TEI and employees’ emotional states fully mediated the relationship between management teams’ intended transformational leadership and employees’ perceived transformational leadership.

**Discussion:**

This study highlights the central role of emotional processes in leadership effectiveness. TEI in management teams enhances the impact of intended transformational leadership (TFL) by shaping followers’ emotional states and perceptions. Positive, high-intensity emotions strengthen perceptions of leadership, whereas low-intensity states, such as comfort, weaken them. These findings advance our understanding of how leaders’ emotional skills and group affect contribute to creating more transformational leadership processes.

## Introduction

Though leadership researchers have long recognized the importance of understanding how leaders are perceived by their followers (i.e., Avolio and Bass, 2004; Craig and Gustafson, 1998; Gessner) and previous studies have examined the relationship between leadership and employee responses (Ertürk et al., 2018; Mindeguia et al., 2021), this investigation will try to shed some light on the specific relationship between the intended and perceived leadership and the mediational effects of group emotional intelligence.

Leadership is a matter of the leader’s actions and the perceptions of followers who interpret what takes place in organizations (Fleenor et al., 2010). Poor congruity between self and other leadership ratings can be a serious problem such leaders may continue to make the same mistakes and be unaware of the issues perceived by workers in their leadership competencies. As Dabke (2016 p.30), mentioned: “…*leaders are not just what they think they are, but also what their followers perceive them as.”*A leader’s influence may depend on followers’ perceptions, thus it is important to analyze the relationship between leaders’ intended leadership styles and followers’ perceptions of such leadership styles. More importantly, if we want to understand how employees interpret a leader’s behavior, it is crucial to analyze the mediation processes that focus on the relationship of these variables.

Numerous leadership styles can potentially impact on workers’ positive responses. Since at least the 1980s, organizational research on transformational leadership has been very popular (Bass, 1985) and it represents one of the most prominent leadership styles. With new leadership models showing very high correlations with transformational leadership, we focus our analysis on the most studied leadership model of the last three decades (Hoch et al., 2018).

Past research had shown that transformational leadership (TFL) accentuates organizational commitment only when a manager intended TFL is coherently perceived by the employees (Jacobsen and Stanoik, 2018). In the same line, it has been found that leadership triggered employee work engagement only when the leader was perceived as practicing TFL (Kopperud et al., 2014). In general, the results of the research in self-other agreement in leadership our results call for more research on both the antecedent and the consequences of perceived leadership.

Moreover, the scarce research on leadership self-other agreement, which analyzes the difference between perceived and intended leadership, has been conducted only at the individual level. Literature about leadership styles has been based mostly on one source study without considering leaders’ and members’ relations (Jacobsen and Andersen, 2015). Leadership is inherently multilevel (Yammarino and Dansereau, 2008), so our understanding of effective leadership will be limited if we fail to integrate individual-level processes with group-level processes (Kozlowski and Bell, 2003). Following Jacobsen and Andersen (2015), the aggregated perception of leadership practices among employees is especially relevant to future leadership studies.

In this sense, Mindeguia et al. (2021) examined the relationship between transformational leadership and employees’ responses to passion and proactive behaviors as mediated by team emotional intelligence. They found that leader teams’ intended leadership style influenced workers’ behavior through their team emotional intelligence and the high-intensity positive emotions of workers. If we consider that the TFL only affects when it is perceived and that TEI and emotions mediate the relationship between intended leadership and results, we could hypothesize that TEI and Passion mediation could explain, in part, the intended-perceived leadership style relation.

Extending this research, this study’s primary objective was to analyze the relationship between the management team’s indented leadership behavior and the work units’ perceived leadership behavior. Moreover, we posit that TEI and emotional states will mediate this relationship.

**Figure 1:**
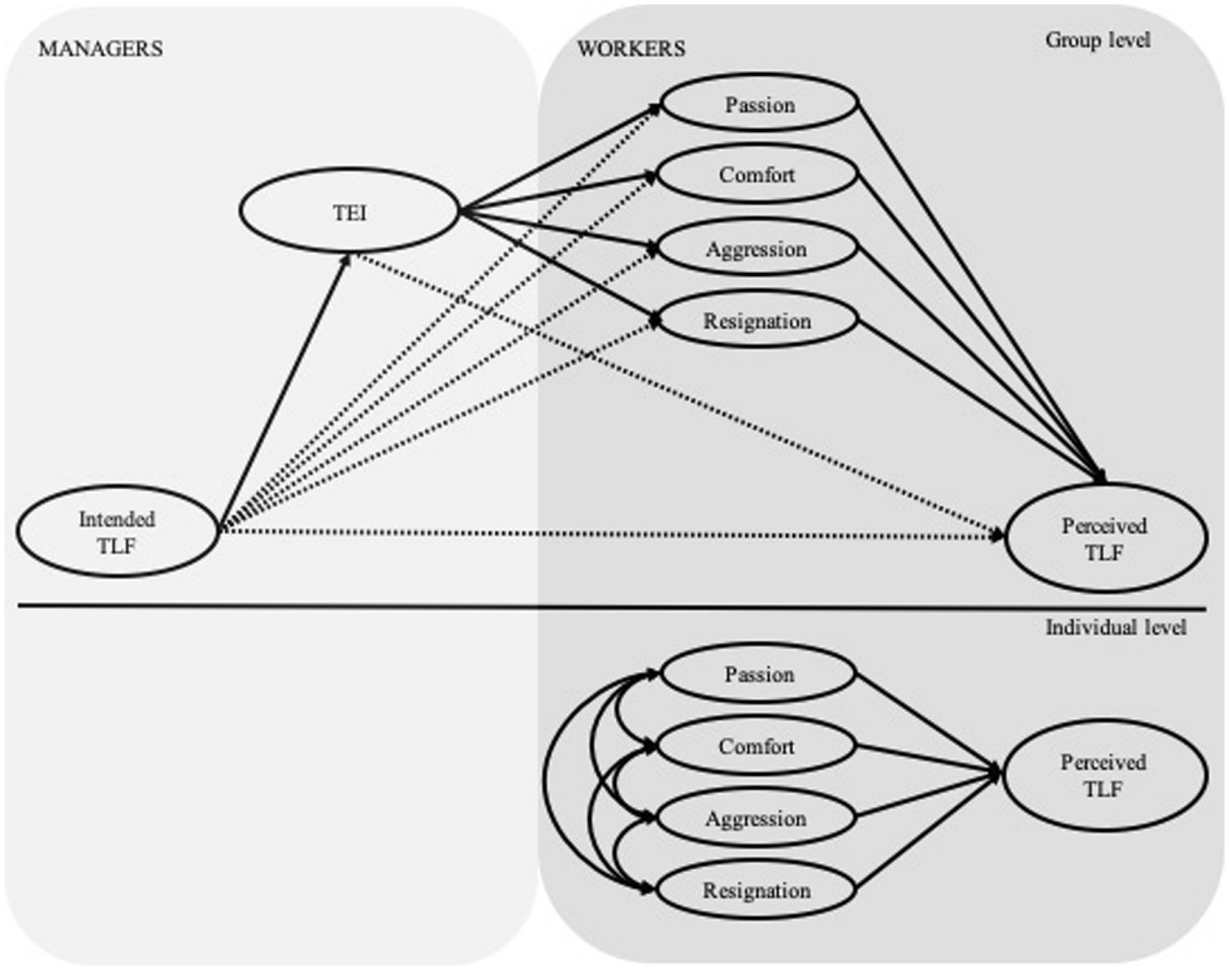
Hypothesized model. TFL, transformational leadership; TEI, team emotional intelligence.

Specifically, this study will analyze the mediating effect of the management team’s EI and the emotional state of the followers’ work units using a multilevel and multisource model. Thus, the hypothesized model (see Figure 1) will propose a mediation model with two mediators (TEI and affective state) and two information sources (leader teams and workers), considering both individual and group levels of analysis (workers and work units).

In doing so, this study research adds new insights beyond the intended and perceived leadership process helping both, academics, and practitioners, to understand what is underlapping this process. This could be helpful when creating new training and intervention sessions for leaders who want to be perceived as transformational. Taking into account that we will use the multilevel analysis methodology, this study also answered the call for a multilevel study of EI (Ashkanasy, 2003; Troth et al., 2017), the affective state (Van Knippenberg et al., 2008), and TFL (Tse et al., 2018).

### Hypothesis development

#### Intended and perceived leadership style

Leadership is defined as a social influence process through which a leader affects subordinates’ feelings, perceptions, and behaviors (Pirola-Merlo et al., 2002). Leadership action is difficult to observe, therefore, it is usually measured by asking leaders, employees, or other actors about their perceptions. The HRM literature argues that intended, actual, and perceived practices are separate but linked concepts (Wright and Nishii, 2007).

Intended practices are those that decision-makers believe will effectively elicit the employee’s desired responses, actual practices are what leaders implement and, finally, the perceived practices are those that are perceived and interpreted subjectively by each employee (Paauwe and Boselie, 2005; Wright and Nishii, 2007).

The research in this vein has shown that the relationships between intended and perceived leadership practices are weak. Many leaders see their leadership as more active than their employees (Jacobsen and Andersen, 2015) as those leaders evaluate their behavior and we expect that they will be ambitious about the behaviors they actually have (Wright and Nishii, 2007).

As mentioned before, TFL is one of the most studied leadership styles and research has demonstrated that it improves employee performance and motivates them to achieve beyond expectations and obligations (Edú-Valsania et al., 2016). TFL is based on four primary behaviors: (1) inspirational motivation, (2) idealized influence, (3) intellectual stimulation, and (4) individualized consideration (Bass, 1985). Transformational leaders, communicate enthusiasm and vision, have a positive outlook, use intuitive insight, and exhibit emotional competency.

As transformational leadership is recognized to be more positive for performance the leaders tend to overrate themself even more for this specific leadership style. In this sense, Jacobsen and Andersen (2015) found that leaders tend to overrate their use of a given type of leadership relative to their employees’ assessments, particularly for TFL.

At the individual level, Lee and Carpenter’s (2018) meta-analysis found that leader and observer ratings of TFL behavior were positively and moderately correlated, ranging between 0.26 and 0.52. From a multilevel point of view, collective perceptions of followers and superiors offer more information about leadership dynamics than focusing only on an effective leader and collecting self-perceptions of one’s leadership behavior (Dabke, 2016).

At the group level, little is known about the fundamental relationship between leader and observer perceptions of leadership. *TFL behavior* communicates the importance of group goals, develops shared values and beliefs among followers, and inspires unified effort to achieve group goals (Wang and Howell, 2010). The influence target is the whole group, meaning that the leader exhibits similar behavior toward different members of the group (Yammarino and Bass, 1990).

Social Identity Leadership Theory (Hogg, 2001; van Knippenberg and Hogg, 2003) proposes that when group members identify with their group and group membership becomes more salient in their self-concepts, leadership effectiveness is then contingent on the extent to which the leader is perceived by followers as a prototypical member of the group.

Following this line, as the group evaluation of the leader would be based on the combination of workers’ individual perceptions, we expect that at the group level, the association between intended and perceived TFL will be positive but with a moderate correlation.

*H1*: Leader teams’ intended TFL will be positively related to followers’ units’ perceived TFL.

Following Jacobsen and Andersen (2015) we can see that some leaders’ leadership intentions are better aligned with employees’ perceptions of leadership than others, but we still know relatively little about why, therefore it becomes crucial to analyze the mediation processes between both concepts.

Following Mindeguia et al. model of the mediation process between intended transformational leadership and the effect on workers, we posit that TEI and the emotional states of workers, will mediate the relationship between perceived and intended TFL.

#### Leadership and affect

Scholars have pointed out the ubiquity of emotion in teams and its influence on team processes (Barsade and Gibson, 2012; Menges and Kilduff, 2015). Based on Affective Events Theory (AET) (Weiss, 2002; Weiss and Cropanzano, 1996), leaders are viewed as critical organizational players who, via their behavior (e.g., giving feedback, allocating tasks, etc.) and mood (e.g., enthusiastic, excited, angry, distressed, etc.) trigger affective events that have consequences for employees and teams (Ashkanasy and Dorris, 2017).

Research on emotions in organizations has shown the effect of different emotional constructs on all organizational levels. Ashkanasy and Tse (2000) proposed that affect is central to developing and maintaining leader-member exchange processes (Tse et al., 2018). In this sense, a growing body of study has demonstrated that emotional intelligence (EI) is an underlying factor associated with leaders’ behavioral styles (Harms and Credé, 2010; Foster and Roche, 2014). Emotionally intelligent leaders assume the role of “emotional managers” to establish a positive “affective tone” (Pescosolido, 2002) for their subordinates’ benefit and to create positive, affective events for them. Through these processes, members are likely to feel more positive and to offer more positive evaluations of the leaders (Diener et al., 2020). This, in turn, increases their respect and admiration for them.

##### TEI as a mediator between intended and perceived TFL

At the individual level, EI’s emotional self-awareness aspect is a critical variable in self-other agreement research (Gardner et al., 2005). Self-aware managers are more responsive to employee perceptions of leadership (Jacobsen and Staniok, 2018). Therefore, they can adapt their behavior to the organization’s requirements, become more effective leaders and be evaluated as more transformational.

Emotionally intelligent individuals positively express their emotions and, thereby, maintain favorable interpersonal relationships at work (Stephens and Carmeli, 2016). In this sense, a growing body of studies has demonstrated that EI is an underlying factor associated with leaders’ behavioral styles (Harms and Credé, 2010; Foster and Roche, 2014). Furthermore, previous studies’ findings show that leaders who scored highly on EI have more options to behave as transformational leaders (Lopez-Zafra et al., 2017). This finding demonstrates a close relationship between both constructs. Also, a recent study showed that leaders ´ TFL had a significant effect on employee engagement for the mediating role of EI (Milhem et al., 2019).

At the team level, Druskat and Wolff (2001) define TEI as *“the ability of a group to develop a set of norms that manage emotional processes”* (Druskat and Wolff, 2001: 133). This set of norms or expected behaviors is generated through subjective emotional experiences that group members share and will define subsequent emotional experiences (Wolff et al., 2006). TEI has been identified as a fundamental source of variability for several variables related to individual and group behavior (Aritzeta et al., 2015; Druskat et al., 2017). TEI has been demonstrated to be a fundamental construct at the team level to improve team understanding of their environment (Druskat et al., 2017). For example, previous studies have shown that emotionally intelligent teams create positive moods in their workplace (Ashkanasy and Dorris, 2017) and reduce emotion-related issues such as stress and burnout. This stress reduction, in turn, leads to heightened team performance (Greenidge and Coyne, 2014).

Druskat and Wolff (2001) stated that TFL behaviors help generate emotionally competent norms leading to higher TEI. When leaders influence the processes, actions, norms, and climate within work teams (Tseng and Levy, 2019), their personalities may affect the team’s emerging character (Lopez-Kidwell et al., 2018). Being part of a work team implies a complex combination of information processing and emotional responding that could influence team members’ responses. The same worker may experience different emotional reactions to a dramatic event on two different teams, depending, for example, on that team member’s leadership style and on how TEI influences their perceptions and behaviors (Ghuman, 2016). In this sense, Mindeguia et al. suggested that management teams composed of transformational leaders have higher TEI and generate more positive emotions in their followers, who then experience greater cohesion within the team.

Teams with high TEI levels cooperate more, coordinate more efficiently on their work, and communicate more effectively than those with low EI (Lee and Wong, 2019). Also, TEI may lead to stronger relationships with co-workers (Jordan and Troth, 2004), better information exchange and decision-making (Ghuman, 2016), and reduced team conflict (Jordan and Troth, 2004).

TEI is associated with better organizational-emotional understanding (understanding the emotional state and need of the organization) and promotes the management of emotions when the group deals with individuals and groups outside of the group’s boundary (Koman and Wolff, 2008). High TEI teams are more adept at appropriately responding to their followers’ emotions (Chang et al., 2011). Moreover, TEI is connected to leadership emergence, the performance of effective leadership behaviors, and overall leadership effectiveness (Lee and Wong, 2019).

Following this rationale, this study posits that the TEI of the management team will mediate the relationship between perceived and intended TFL; in the sense that teams showing higher TEI will be perceived as more transformational by their followers than those with lower levels of TEI.

*H2*: TEI will mediate the relationship between leaders’ intended TFL and followers’ perceived TFL in the sense that intended TFL will be positively related to TEI, and TEI will be positively associated with perceived TFL.

##### Affect and perceived TFL

Research has shown that one of the most critical variables that affect workers’ perception and judgment is their affective states (Ashkanasy and Dorris, 2017). Circumflex models of emotions (Russell, 1980) have proved to be useful for explaining the relationship between leadership, affect, and emotions (Peñalver et al., 2017). In addition to measuring the effect of positive and negative emotion based on the quality of emotion (pleasure vs. displeasure), this model proposed another dimension: activation (pleasure vs. displeasure). Thus, while some positive feelings are activating (e.g., excited, enthusiastic), others are deactivating (e.g., calm, relaxed). Similarly, some emotions are negative in tone with high activation (e.g., anxious, angry), while other negative feelings are deactivating (e.g., discouraged, bored). Following Bruch and Ghoshal (2003), the intersection of these two dimensions of quality and activation determines four potential affective states: (1) comfort (pleasure and low activation), (2) resignation (displeasure and low activation), (3) passion (pleasure and high activation), or (4) aggression (displeasure and high activation).

While affective valence has traditionally been regarded as the more influential dimension of job-related affect (e.g., see Fisher, 2010 for a review), more recent evidence (To et al., 2015; Peñalver et al., 2017) suggests that affective activation also plays an essential role in motivating job behaviors.

Following the meta-analysis of Madrid and Patterson (2018), experimental results confirmed that people in a negative mood tended to make more critical, self-deprecating interpretations and attributions. Those in a positive mood selectively looked for and found lenient and optimistic explanations for identical outcomes.

Specifically, low-activated positive affect (in this research, comfort) is expressed, for instance, in feelings like tranquility and calmness, which inform individuals that the environment is free of threats that might compromise performance (Peñalver et al., 2017). Broadening cognition is predominantly expressed in an open attentional focus and top-down flexible and divergent ways of thinking (Diener et al., 2020). These psychological processes should be less associated with problem identification (Madrid and Patterson, 2018). Further, high-activated positive affect (passion, in this research) entails feelings such as enthusiasm, joy, and inspiration. These are linked to the perception of successful task performance opportunities such as expanded attentional focus and flexible information processing manifested in divergent thinking (García-Buades et al., 2020). These positive emotion zones may affect worker perceptions, increasing their positive evaluations of the leaders.

Furthermore, negative affect can create social distance by increasing competition or motivating people to withdraw from social interactions (Meng et al., 2015). High-activated, negative affect (aggression) involves unpleasant and energized feelings, such as anxiety, tension, and worry. These feelings are associated with appraisals about hazards and problems to be solved in the environment, such as threats to work performance (Knight and Eisenkraft, 2015). Low-activated, negative affect (resignation), characterized by feelings such as depression, dejection, and despondency, signal that something is wrong in the environment and are typically associated with the experience of loss or failure to achieve the desired outcome (Gable and Harmon-Jones, 2010; Treynor et al., 2003). The limited activation embedded in these feelings leads to disengagement with the environment, social apathy, and passiveness (Verhaeghen et al., 2005).

Based on the aforementioned theory, we hypothesized that at the individual level emotional state shown by a worker is related to TFL perception in the sense that:

*H3.a*: At the individual level, the passion emotional state will be positively related to the perception of TFL.

*H3.b*: At the individual level, the comfort emotional state will be positively related to the perception of TFL.

*H3.c*: At the individual level, the resignation emotional state will be negatively related to the perception of TFL.

*H3.d*: At the individual level, the aggression emotional state will be negatively related to the perception of TFL.

At the group level, Kelly and Spoor (2007) concluded that the effects of individual moods could be extended to the team level. Similarly, at the team level, members’ shared moods might also influence their team’s motivational (e.g., team goal commitment), attitudinal (e.g., team satisfaction), and behavioral (e.g., the team helping behaviors) processes over a specific period of time (George and King, 2007; Kelly and Spoor, 2007).

In addition, studies have shown that emotionally intelligent teams create positive moods in their workplace (Ashkanasy and Dorris, 2017) and, for example, reduce stress and burnout, which in turn lead to heightened TFL perception (Greenidge and Coyne, 2014). Leader teams with high TEI should be able to transmit their emotions via emotional contagion mechanisms to lift their followers’ positive feelings and satisfaction levels (Ilies et al., 2013).

Considering the influence of TEI on the affective responses of workers and the effect of affect in judgment and perception, we state that: At the group level, emotional states will mediate the relationship between the intended TFL of the management team, the management teams’ EI, and the followers’ perceived TFL. Specifically, we hypothesize that:

*H4.a*: The passion emotional state will mediate the relationship between intended TFL, TEI, and followers’ perceived TFL.

*H4.b*: The comfort emotional state will mediate the relationship between intended TFL, TEI, and followers’ perceived TFL.

*H4.c*: The aggression emotional state will mediate the relationship between intended TFL, TEI, and followers’ perceived TFL.

*H4.d*: The resignation emotional state will mediate the relationship between intended TFL, TEI, and followers’ perceived TFL.

In summary, the hypothesized model posits that, at the individual level, workers’ emotional states of passion and comfort will have a positive relationship with TFL perception. Similarly, aggression and resignation will have a negative association with TFL perception.

At the group level, we posit that the TFL behaviors of the management team will help to generate TEI. Simultaneously, emotionally intelligent leader teams will act as emotional managers, eliciting more positive and less negative emotions in worker units to evaluate those leaders more favorably.

## Method

### Participants

Data for this study were gathered in 2018 from two sources: (1) 186 leader teams composed of 1,550 leaders and (2) 4,561 workers grouped into 186 business areas in which the leaders reside. In the total sample, 38% of the participants were female, and the average age was 42 years (SD = 8.68).

The organizations participating in this study were settled in the Basque Country (northern Spain). All the firms are part of the well-known Mondragon Cooperative Corporation, which shares four corporate values: (1) cooperation, (2) participation, (3) social responsibility, and (4) innovation. The organization is distributed along different economic sectors: industry (*N* = 30; 33.3%), service sector (*N* = 22; 24.4%), education (*N* = 7; 7.8%), and distribution (*N* = 31; 34.4%). Further, 47.8% (*N* = 43) are small organizations, 40% (*N* = 36) are medium-sized organizations, and 12.2% (*N* = 11) are large organizations.

### Procedures

In this study, data were collected after directors of the participating firms agreed upon the study conditions. The questionnaires were distributed by two means randomly assigned to participants: (1) online questionnaire and (2) paper-and-pencil (hard copy). For those who responded online, the questionnaire was sent via email. For those who completed the paper-and-pencil version, employees gathered in a large meeting room with the help of the human resources (HR) director to assure anonymity. The ethics requirements established by data protection regulations were met, and the University of Mondragon’s ethics committee approved the study. No differences were found between these two groups of respondents.

The data obtained was incorporated into a file for statistical treatment. Data were analyzed using IBM SPSS 24 and MPLUS 7 statistical software. The leaders’ data were aggregated and merged with worker data using the organizational area as a critical variable.

### Measures

#### Individual-level measure

##### Emotional states

The four dimensions for this construct were extracted from Russell’s theoretical classification of emotions (Russell, 1980). The aggression dimension was composed of four emotions (i.e., “In my work I usually feel furious”), passion with four emotions (i.e., “In my work I usually feel enthusiastic”), resignation with six emotions (i.e., “In my work I usually feel discouraged”), and comfort with three emotions (i.e., “In my work I usually feel comfortable”). The Cronbach’s Alphas obtained in this study were 0.89 for resignation, 0.82 for passion, 0.87 for aggression, and 0.68 for comfort.

##### Perceived transformational leadership

Three dimensions of perceived TFL, namely vision, positive leadership, and supportive leadership, were measured using the scale developed by Rafferty and Griffin (2006). For example: “*My supervisor has a clear understanding of where we want our unit to be in 5 years.”* The three dimensions were operationalized by three items each and showed excellent internal consistency (Cronbach’s alpha of 0.85 for vision, 0.92 for positive leadership, and 0.93 for supportive leadership). The Organizational Culture Inventory (OCI) (Cooke and Lafferty, 1983) was used to measure the leadership goal emphasis dimension. The dimension, composed of three items, had a Cronbach’s alpha of 0.86.

#### Group level measures

##### Team emotional intelligence scale

T-TMMS (Team-Trait Meta Mood Scale; Aritzeta et al., 2020) was utilized to assess the TEI. The T-TMMS is a self-reporting questionnaire that measures the level at which leaders of the same team (reference group) pay attention to and value teammates’ feelings. It measures whether the team’s emotions are clear or confused and whether leaders use positive thinking to repair the team’s negative moods. For example: “*We usually know what we feel in different situations.*” The Cronbach’s alpha values for the three dimensions of T-TMMS (three items for each one) were 0.76 for attention, 0.80 for clarity, and 0.88 for repair.

##### Transformational leadership

The scale of group perception of exercised leadership was adapted (changing point of reference from individual self-perception to group self-perception) from two sources:

- The Rafferty and Griffin (2006) scale for the vision (LV), positive leadership (LP), and supportive leadership (LS) dimensions.
- The Organizational Culture Inventory (OCI) by Cooke and Lafferty (1983) for goal emphasis dimension. For example: *“We have a clear understanding of where we want our unit to be in 5 years.”*

Confirmatory Factor Analysis was conducted to confirm the factorial structure of the new scale. The model showed a good fit (χ^2^df = 227.48, p.0001, CFI = 0.97, TLI = 0.96, RMSEA = 0.06, 90%) with adequate factor loadings, and replicated the original scale with four dimensions. The dimensions (LV, LP, LS, LG) had a Cronbach’s alpha of 0.85, 0.92, 0.93, and 0.86, respectively.

## Results

### Descriptive statistics and aggregation indices

To determine if aggregating individual responses to team-level constructs is adequate, we followed the procedure described by Van Mierlo et al. (2009). That procedure includes the examination of *rwg* and *ICC1* and 2. The *rwg* values are a measure of agreement within the group. *ICC1* is the proportion of variance in ratings due to team membership, and *ICC2* is the reliability of team mean differences (Klein et al., 2000). Bliese (2000) concluded that *ICC1* values exceeding.05 are sufficient to warrant aggregation. LeBreton and Senter (2008) suggested cut-off values that range from 0.70 to 0.85 for *ICC2.* Also, they concluded that *rwg* values between 0.51 and 0.70 indicate moderate agreement; *rwg* values between 0.71 and 0.90 show strong agreement, and *rwg* values between 0.91 and 1.0 indicate strong agreement.

For perceived TLI dimensions, *ICC1* values were between 0.14 and 0.23, between 0.80 and 0.87 for *ICC2,* and between 0.69 and 0.70 for *rwg*. For emotional states, the *ICC1* values were between 0.09 and 0.16; the *ICC2* values were between 0.70 and 0.82, and *rwg* was between 0.70 and 0.80; the resignation zone was the only exception. The *IC1* for resignation was 0.06 and 0.62 for *ICC2.* Therefore, we cannot consider resignation as a group variable. Even so, we concluded that the *ICC1*, *ICC2,* and *rwg* indices justified the aggregation of individual variables in the remaining cases.

The descriptive statistics for all variables, including the mean, standard deviations, and bivariate correlations between the variables, are presented in Table 1.

**Table 1:** Descriptive statistics.

Variables	Mean (sd) individual	Mean (sd) group	1	2	3	4	5	6	7	
1. INTENDED TFL	-	4.60 (0.48)	1	0.62**	0.17*	−0.18*	0.02	-	0.44**	Group level
2. TEI	-	4.45 (0.50)	-	1	0.24**	0.21**	0.11*	-	0.43**	
3. PASION	4.25 (1.04)	4.31 (0.49)	-	-	1	−0.57**	0.53**	-	0.49**	
4. AGRESION	2.33 (1.10)	2.32 (0.47)	-	-	−0.40**	1	−0.32*	-	−0.40**	
5. CONFORT	3.59 (1.10)	3.63 (0.47)	-	-	0.51**	−0.33**	1	-	0.17*	
6. RESIGNATION	1.93 (0.95)	-	-	-	−0.39**	0.67**	0.32**	1	−0.33**	
7. PERCEIVED TFL	3.88 (1.14)	3.98 (0.64)	-	-	0.46**	0.36**	0.25**	−0.31^**^	1	
**p < 0,01; *p < 0,05.			Individual level							

TFL, transformational leadership; TEI, team emotional intelligence.

In this sense, Hypothesis 1 posited that the intended TFL of the management team would be positively and moderately related to the perceived TFL of the work units. Based on Cohen’s (2013) benchmarks of effect sizes, correlations greater than 0.52 would indicate strong agreement. Correlations ranging between 0.26 and 0.52 would reflect a moderate level of agreement; correlations below 0.26 would demonstrate a low agreement level. Thus, the descriptive results confirmed Hypothesis 1.

### Hypotheses testing

**Figure 2:**
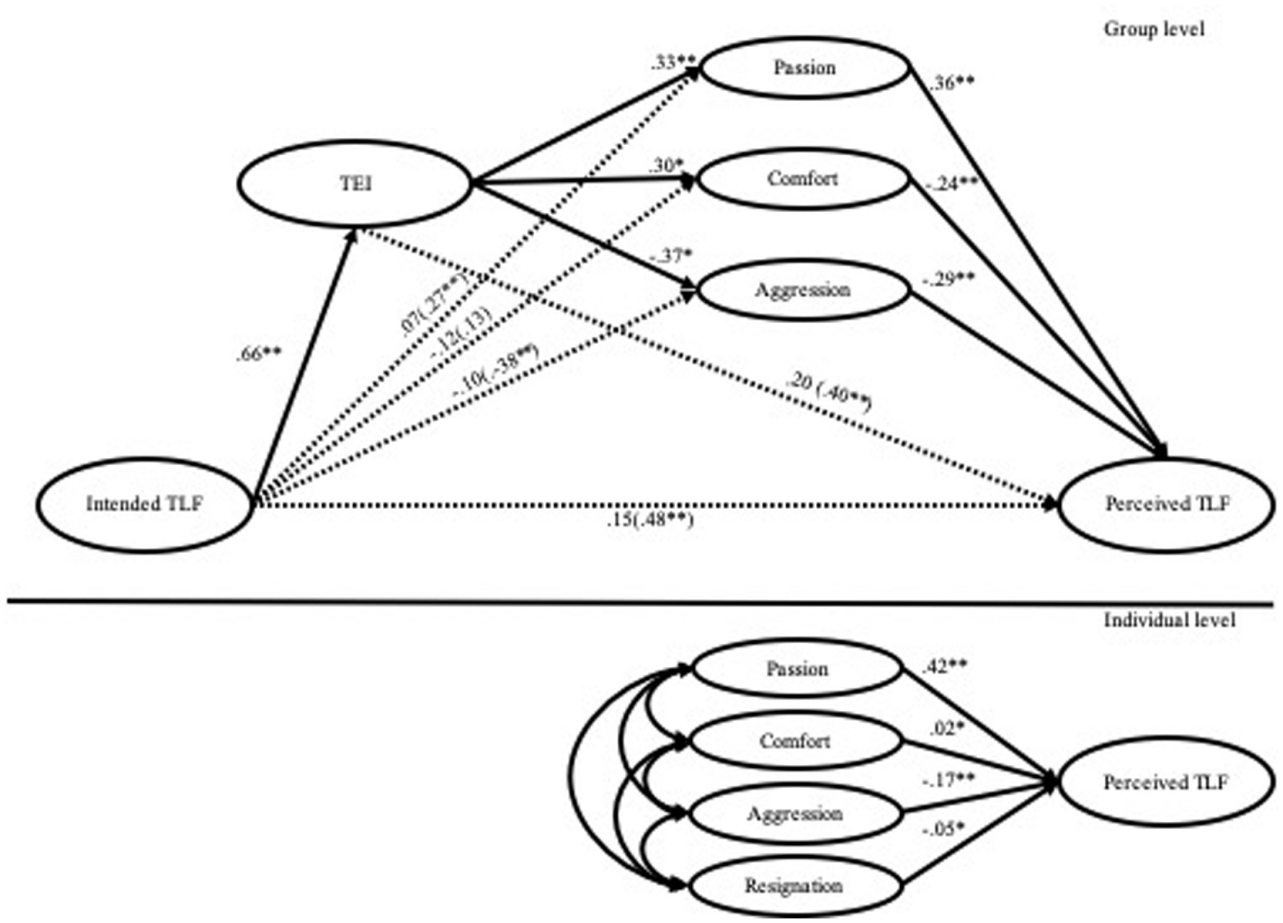
Model results. TFL, transformational leadership; TEI, team emotional intelligence.

To test Hypothesis 2, we conducted a multilevel path analysis with Mplus. The results are presented in Figure 2. The model fit indexes (CFI = 0.97; TLI = 0.96; RMSEA = 0.03) demonstrate the excellent fit of the analyzed model.

In support of Hypothesis 2, TFL was positively related to TEI at the group level (*β* = 0.66, *p* < 0.01), and TEI was positively associated with passion (*β* = 0.33, *p* < 0.01) and comfort (*β* = 0.30, *p* < 0.05) but negatively related to aggression (*β* = −0.37, *p* < 0.05). No association to resignation was detected due to insufficient agreement at the group level.

Hypotheses 3a to H3.d were supported. At the individual level, both passion and comfort were positively related to TFL perception (*β* = 0.42, *p* < 0.01; *β* = 0.02, *p* < 0.05). In the same line, aggression and resignation were negatively related to TFL perception (*β* = −0.17, *p* < 0.01; *β* = 0.05, *p* < 0.05).

At the group level, Hypothesis 4.a stated that passion emotional state would mediate the relationship between intended TFL, TEI, and followers’ perceived TFL. In this sense, passion was positively related to TFL perception (*β* = 0.36, *p* < 0.01). Therefore, Hypothesis 4.a was supported. Hypothesis 4.b (comfort emotional state) would mediate the relationship between intended TFL, TEI, and followers’ perceived TFL. Nevertheless, comfort was negatively related to TFL perception (*β* = −0.24, *p* < 0.01); thus, Hypothesis 4.b was not supported. Hypothesis 4.c (aggression emotional state) would mediate the relationship between intended TFL, TEI, and followers’ perceived TFL. The results support our hypothesis that, at the group level, aggression was demonstrated to be negatively related to leader TFL perception (*β* = −0.29, *p* < 0.01). Finally, Hypothesis 4.d was not supported; resignation was not identified as a group-level construct due to insufficient agreement at the group level.

To confirm the mediation processes, direct and indirect effects were estimated. Direct effects are presented in Figure 2, and indirect effects are shown in Table 2. There was a full mediation effect of TEI in the relationship between TFL and emotional states. The emotional states of passion, aggression, and resignation mediate the relationship between TEI and TFL perception. Nevertheless, only one indirect effect proved to be significant, the indirect impact through passion. Thus, we concluded that the mediation model was only supported through high-intensity positive emotions and TEI.

**Table 2:** Indirect effects.

	Indirect effect	*p*-value
TFL-TEI- PTFL	0.20	0.06
TFL-PASSION- PTFL	0.04	0.63
TFL-CONFORT- PTFL	0.04	0.50
TFL-AGGRESSION- PTFL	0.04	0.50
TFL-TEI-PASSION- PTFL	0.11	0.03
TFL-TEI-CONFORT- PTFL	−0.07	0.20
TFL-TEI-AGRESSION- PTFL	0.10	0.06

TFL, transformational leadership; PTFL, perceived transformational leadership; TEI, team emotional intelligence.

## Discussion

Leadership is a process of influence that only has an effect if it is perceived (Dabke, 2016) so measuring the workers’ perception of the leadership has been demonstrated to be relevant. This study aimed to address the relationship between TFL as perceived by the subordinate, the TEI, the affective states of followers, and TFL behavior of the managerial team. Previous research showed that the effectiveness of TFL is based on the perception of this behavior by the followers; thus, it becomes significant to understand better how the intended TFL and the perceived TFL are related.

By examining emotions and their influence on perceived TFL from a multilevel perspective, we integrate the individual and team levels in emotions research. In particular, our findings show how understanding and managing emotions is a central part of leadership effectiveness. Incorporating the aforementioned levels in one study may be essential because it should contribute to each of the theoretical domains of group affect and leadership by answering questions concerning how and why TFL and TEI enhance leadership effectiveness (Tse et al., 2018). Moreover, our study also answers, from a emotional point of view, the research question proposed by Jacobsen and Andersen (2015) about why some leaders’ leadership intentions are better aligned with employees’ perceptions of leadership than others.

The results supported our predictions: TFL behaviors are positively related to the perception of TFL (Hypothesis 1) workers have. Nevertheless, the effect was moderate. Moreover, the relationship between intended and perceived TFL was mediated by TEI (Hypothesis 2) and the passion emotional state (Hypothesis 4).

Specifically, we found that (1) TFL behaviors are positively associated with higher levels of TEI in the management teams, (2) the TEI of the leadership teams fully mediates the relationship between TFL behaviors of the leadership teams and the emotional or affective states of the subordinates at the team level, (3) the affective states mediated the relationship between TEI of the leadership team and the perception of the TFL by subordinates, and (4) at the individual level, all the affective states influenced the perception of the TFL held by subordinates.

At the individual level, the positive-balanced emotional states showed a positive association with the perception that TFL employees had individually. In contrast, negative-balanced emotional states showed negative attitudes with that perception. High-intensity emotional states showed a stronger relationship with perceived TFL. This is consistent with the argument that workers who experience positive moods are more likely to offer positive evaluations of their leaders (Bono and Ilies, 2006).

To analyze the mediating role of TEI at the group level, this study added knowledge to the theory on leadership effectiveness and provides evidence of the importance of emotions in organizations. Our results are consistent with research conducted at the individual level, which found that managers with high EI are more aware of their follower’s perceptions of their leadership. Therefore, they can adapt their behavior to the organization’s requirements to become more effective leaders and being evaluated as more transformational (Jacobsen and Staniok, 2018). Following Tepper et al. (2018), the effectiveness of TFL is based on the fit between the subordinates’ need for that behavior and the received TFL. In this sense, teams with high TEI are more adept at appropriately recognizing and responding to their followers’ emotions and needs (George, 2000; Chang et al., 2011).

Leaders’ proper response to followers is related to more positive and less negative emotional states. The AET supports this finding. Through their behavior (e.g., giving feedback, allocating tasks, etc.) and moods (e.g., enthusiastic, excited, angry, distressed, etc.) leaders, influence workers’ well-being. Leaders take the role of “emotional managers” to establish a positive “affective tone” (Pescosolido, 2002) between their subordinates create positive affective events for them. Through these processes, workers are likely to feel more positive and to offer more positive evaluations of their leaders (Diener et al., 2020).

Nevertheless, positive emotions are not always linked to positive outcomes (e.g., hubristic pride), and negative emotions are not always related to adverse consequences (e.g., anger motivating an individual to respond to a social justice problem) (Lindebaum et al., 2017). In this sense, the comfort emotional state is defined by low intensity and a relatively positive level of valence with positive emotions such as calm and contentedness. The emotional state’s low intensity does not spur people to action, and companies in the comfort and resignation zones operate at low levels of attention, emotion, and activity (Bruch and Ghoshal, 2003). The low intensity can explain the negative relation between the comfort state and the TFL perception at the group level.

In addition, it appears that low-intensity affective states were not as contagious as high-intensity emotional states (as can be seen by the ICC indices of our results). Moreover, the emotional contagion of negative affective states is less than positive. In this sense, research has shown that unpleasant emotions may not spread as expected because of the non-normative nature of unpleasant emotions (Stephens and Carmeli, 2016). People with low energy (low activation or intensity) and unpleasant affect are typically less socially oriented (Barsade, 2002). Therefore, they become more internally oriented and withdrawn from the group, resulting in fewer opportunities to influence them (Barsade, 2002).

Finally, in analyzing the overall model, we concluded that management teams that perceive themselves as TFL have more TEI and transmit more positive (and less negative) emotions to their followers, who perceive them as more transformational. The evidence presented shows the importance of TEI and emotional states in the relationship between leaders and subordinates. Accordingly, we concluded that emotionally intelligent teams have a greater understanding of their employees’ needs and are more effective in responding to them.

Studies conducted in diverse organizational settings indicate that leadership marked by high emotional intelligence is linked to enhanced team emotional intelligence and improved team outcomes. Lam and O’Higgins (2015) report that transformational leadership serves as a mediator between leaders’ emotional intelligence and benefits such as greater team effectiveness, improved communication, and better conflict management. Gulzar and Rehman (2021) note that ethical leadership bolstered by emotional intelligence strengthens team effectiveness.

Additional results shed light on several mechanisms that underpin the relationship between emotional intelligence and leadership outcomes. Transformational leadership behavior, as shown in multiple studies, serves as a key link between leaders’ emotional intelligence and favorable team outcomes. Emotional processes at the team level—such as emotional contagion and the development of emotionally competent group norms (Mindeguia et al., 2021)—further explain how a leader’s emotional intelligence translates into improved performance, trust, and commitment. Furthermore, positive associations between leader emotional intelligence and outcomes like employee cohesion and business results have been found across various sectors, including healthcare, call centers, military, and manufacturing.

These findings support the view that emotionally intelligent leadership enhances emotional competencies and fosters beneficial team outcomes. The emerging field of meaningful leadership (Frémeaux and Pavageau, 2020; Batuchina et al., 2025) adds another dimension to understanding leadership effectiveness. Meaningful leadership, which emphasizes purpose, values, and the deeper significance of work, may intersect with team emotional intelligence (TEI) by enhancing leaders’ ability to connect with and motivate their teams. Future research could explore how meaningful leadership complements or enhances the impact of TEI in shaping perceptions of transformational leadership.

### Practical implications

Our study has several practical implications. First, our research emphasized the importance of emotions and affectivity at both individual and team levels. Following Jacobsen and Andersen (2015), to provide a clear recommendation for leaders, we must better understand the potential for affecting perceived leadership. Our results showed that the emotional constructs such as TEI and followers’ emotional responses mediate the relationship between what the leaders think they do and how their behavior is perceived. Therefore, this study demonstrated the importance of generating emotional skills in the workplace.

Our findings reaffirmed that managers must consider TEI as an essential skill and a prerequisite criterion in hiring, promoting, and training project leaders, managers, and project teams. The evidence presented can be used to promote workers’ well-being and create emotionally healthier organizations. Activities aimed at increasing leaders’ teams’ EI would indirectly impact workers’ well-being and organizational well-being. Prior research on large projects found that training can improve project team members’ EI (Clarke, 2010).

Moreover, if leaders want to be perceived as more transformational, they should train their emotional intelligence in order to be capable to understand better their follower needs. Additionally, future leadership development programs might benefit from incorporating principles related with emotional intelligence, which could further enhance leaders’ capacity to inspire and engage employees. Furthermore, organizations should explore tailored leadership training programs that integrate TEI related with concepts as meaningful leadership to foster a work environment that maximizes both leader effectiveness and employee motivation.

### Limitations and future directions

The current study, however, was limited in some respects. We identified four such limitations. First, the results were based on self-reported data, which may partially hide real answers. Further, the study should have provided more objective measures for verifying the impact of TEI on organizations.

Foremost, it will be necessary to examine further EI and its relationship to performance in different cultural contexts and other projects. The single organizational context in which we examined the hypothesized relationships limited our ability to generalize our findings.

Additionally, incorporating the emerging concept of meaningful leadership into future research could provide further insights into how leaders can enhance both their own effectiveness and their teams’ emotional engagement. Exploring the intersection of meaningful leadership and TEI in various organizational settings would be particularly valuable in expanding the understanding of leadership effectiveness. Moreover, future research could assess the effectiveness of structured interventions that simultaneously develop TEI and positive ways of leadership to determine their impact on leadership perceptions and overall organizational outcomes.

Finally, this study did not consider EI’s dynamic nature in the workplace because we did not collect longitudinal or qualitative data. Therefore, we were unable to draw causal conclusions from our study.

Nevertheless, this study provides empirical results and amplifies the knowledge about the effect of emotions in organizations and effective leadership. Future investigations should explore this relationship in a variety of organizational contexts.

## Data Availability

The raw data supporting the conclusions of this article will be made available by the authors, without undue reservation.
